# Corrosion of Two Iron-Based Aluminaforming Alloys in NaCl-MgCl_2_ Molten Salts at 600 °C

**DOI:** 10.3390/ma17133224

**Published:** 2024-07-01

**Authors:** Louis Pellicot, Nathalie Gruet, Jérôme Serp, Romain Malacarne, Sophie Bosonnet, Laure Martinelli

**Affiliations:** 1 Université Paris Sorbonne Chimie Physique et Chimie Analytique de Paris-Centre, 75005 Paris, France; 2 Service de Recherche en Corrosion et Comportement des Matériaux, CEA, Université Paris-Saclay, 91190 Gif-sur-Yvette, France; nathalie.gruet@cea.fr (N.G.); romain.malacarne@cea.fr (R.M.); sophie.bosonnet@cea.fr (S.B.); laure.martinelli@cea.fr (L.M.); 3 CEA, DES, ISEC, DMRC, University of Montpellier, Marcoule, 30207 Bagnols sur Ceze, France; jerome.serp@cea.fr

**Keywords:** intergranular oxidation, OC1/OC4 steels, electrochemical techniques, SEM-FIB, predominance diagram, oxo-acidity

## Abstract

Molten salts have been used as heat transfer fluids since the middle of the 20th century. More recently, molten chloride salts have been studied for use in concentrated solar power plants or molten salt reactors. However, none of the materials studied to date has been able to withstand this highly corrosive environment without controlling the salt’s redox potential. The alumina-forming alloy was a promising option, as it has not yet been widely studied. To investigate this possibility, two iron-based alumina-forming alloys were corroded in NaCl-MgCl_2_ eutectic at 600 °C for 500 h after being pre-oxidised to grow a protective layer of α-alumina on each alloy. A salt purification protocol based on salt electrolysis was implemented to ensure comparable and reproducible results. During immersion, alumina was transformed into MgAl_2_O_4,_ as shown by FIB-SEM observation. Inter and intragranular corrosion were observed, with the formation of MgAl_2_O_4_ in the corroded zones. The nature of the oxides was explained by the predominance diagram. Intragranular corrosion was 2 µm deep, and intergranular corrosion 10 µm deep. Alumina formed at the bottom of the intergranular corrosion zones. The depth of intergranular corrosion is consistent with O diffusion control at the grain boundary.

## 1. Introduction

The molten salt reactor (MSR) is a next-generation reactor design in which the coolant is a molten salt [1,2]. CEA Saclay is currently studying corrosion in molten chlorides to develop an MSR that would use NaCl-MgCl_2_-PuCl_3_/Am as a fuel. The salt is then the coolant and the fuel as it contains PuCl_3_ and possibly UCl_3_ [3,4]. This design enables the fuel to be renewed without shutting down the plant by regularly using new batches of salt. It also makes the plant safer than a pressurised water reactor since the reactivity coefficient is negative. In the event of excess reactivity, the temperature rises and the salt expands, increasing the distance between the plutonium atoms and reducing reactivity [5]. The reactor is then self-regulating. Before studying the ternary salt, the binary salt NaCl-MgCl_2_ is used in corrosion tests because it is the fuel solvent.

However, NaCl-MgCl_2_ is a very corrosive medium due to the presence of dissolved impurities such as oxide ions, water, oxygen and chlorine in the salt [6]. These impurities are mainly due to their reactivity with oxygen and moisture, as MgCl_2_ is very hygroscopic [7,8]. In order to have comparable and reproducible data, it is very important to quantify the impurities in the molten salt before any immersion [9]. Various methods have been described in the literature, like cyclic voltammetry, square-wave voltammetry or the acid–base titration [10,11,12]. A purification protocol must be implemented to reach a state where the salt is free of impurities. To reach this objective, the dehydration and melting of NaCl-MgCl_2_ salts must be studied.

In molten salts, two types of alloys can potentially be used: chromia-forming alloys and alumina-forming alloys. Chromia-forming alloys have been widely studied in molten chlorides. They are not suitable for MSR application due to chromia solubility in molten chlorides [13]. Iron-based alloys undergo intragranular corrosion that can be superior from 20 to 30 µm deep after 100 h of immersion [14,15,16,17]. The dissolution of the matrix is often observed with the apparition of intergranular corrosion. MgO has been observed at the surface of the corroded alloys, and Mg–Cr–O oxides have also been observed by different authors [14,18,19]. The same corrosion facies are observed in the corrosion of nickel-based alloys like Inconel 625 [6,18,20]. If the matrix does not dissolve, iron and chromium will preferentially dissolve from the matrix, as reported by Mortavazi et al. [6]. This author also showed that the level of purity of the salt in terms of moisture and oxide ions plays a major role in the behaviour of the immersed alloy: a cleaner salt will be less corrosive.

There are few studies on alumina-forming alloys [21,22,23]. Even if it is difficult to compare different studies that do not provide information on the purity level of their salt, alumina-forming alloys seem to be a better alternative, as shown by Gomez-Vidal et al. [21]. They proved the usefulness of preoxidation to enhance the corrosion resistance of alumina-forming alloys in molten chloride salts. 

The aim of this study is twofold: (i) to design and investigate a purification protocol for NaCl-MgCl_2_ molten salts that will ensure reproducible and comparable corrosion results between different alloys; (ii) to assess whether alumina-forming alloys are attractive candidates as structural materials for MSRs. To this end, for economic reasons, alumina-forming steels were considered in this study (Ni-based alloys will be evaluated in another study).

OC1 and OC4 steels were developed by Oak Ridge National Laboratory (ORNL) in collaboration with Carpenter Technologies [24]. They were designed to be used as micro turbine recuperator components; thus, they were designed to withstand oxidation and creep, after which the alloys were named (OC: Oxidation Creep). Their low nickel content allows them to be economical, and their high Nb content makes them resistant to creep due to the formation of niobium carbides. Their high aluminium content makes them resistant to high-temperature corrosion (up to 200 °C higher than conventional stainless steels) due to the formation of an alumina scale at the surface of the material. They were tested up to 6000 h in engines and showed good performance overall, but a 2–3 µm-deep aluminium depletion was observed under the surface. According to their designers, these alloys are very promising and cost-effective for micro turbine applications but also for all industrial applications up to 700–750 °C. Above 750 °C, OC1 and OC4 are subject to internal aluminium oxidation, which makes them unsuitable for such applications. ORNL is currently studying the effect of minor elements on the ability of alloys to form alumina in order to increase their operating temperature range. A corrosion period of 500 h was chosen because previous studies of the corrosion of alumina-forming alloys have demonstrated the good corrosion resistance of In-702 alloy immersed up to 185 h in MgCl_2_-KCl salt in cycling temperature from 500 °C to 700 °C [25]. This test should enable OC1 and/or OC4 to be qualified for other longer-term tests, notably on corrosion loops. The present work also aims to understand the behaviour of alumina during prolonged immersion in molten chloride, given that Al_2_O_3_ has been reported in the literature to be capable of immersion of 185 h. This study should provide a better understanding of corrosion mechanisms in molten chloride salts in order to select or design an alloy that performs better than OC1 and OC4 in molten NaCl-MgCl_2_.

## 2. Materials and Methods

Molten salt was prepared from NaCl supplied by Sigma-Aldrich with 99.9% purity and MgCl_2_ supplied by Acros International with 99% purity. The salts were stored in a glovebox under pure argon. They were mixed to form approximately 180 g of the eutectic 0.57 mol NaCl—0.43 mol MgCl_2_. FeCl_2_ and CrCl_2_ were supplied by ThermoScientific (Geel, Belgium) with a purity of 99.9%. Both components were anhydrous.

**Figure 1:**
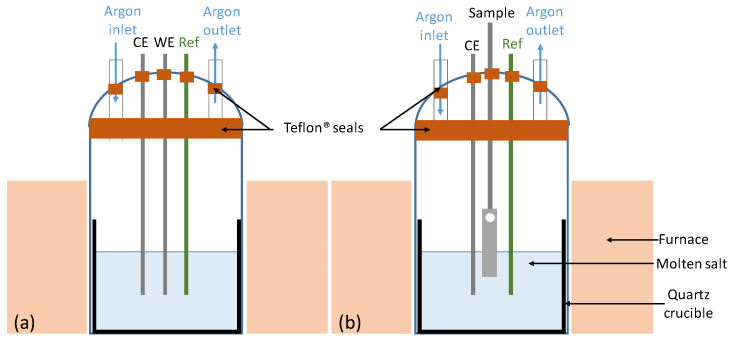
Experimental set-up. (**a**) Electrochemistry. (**b**) Corrosion test.

The experimental set-up is described in Figure 1a,b. The reactor consists of a lower section and a lid through which the electrodes and sample holder pass. The lid and lower section are sealed with a Teflon^®^ gasket (DWK, Meiningen, Germany). High-purity argon (Ar BIP) sweeps the volume above the liquid salt. The working and counter electrodes used are 99% purity, 1 mm diameter tungsten wires supplied by Goodfellow (Lille, France), and the reference electrode is Ag/AgCl. The electrodes consist of a Pyrex tube through which a tungsten wire passes. A silicone paste seals the top of the tube. The reference electrode is custom-made using a silver wire supplied by Thermoscientific (Geel, Belgium) immersed in the 0.55 mol NaCl—0.40 mol MgCl_2_—0.6 mol AgCl salt contained in a Pyrex pocket.

Cyclic voltammetry (CV) was mainly used to analyse the molten salt after experiments. A preliminary parametric study aiming at defining the optimal potential sweep rate was carried out between 20 mV/s and 200 mV/s on the tungsten/molten chloride salts system and led to the choice of 100 mV/s. This study allowed us to choose a sweep rate that gives a quick and stable response as well as a good definition of peaks.

The working electrode surface was 0.33 cm^2^. All electrochemical experiments were performed using an SP-200 potentiostat (Biologic, Seysinnet-Pariset, France) controlled by an EC-lab software v11.43. The voltammograms were traced 5 times, and the 4th cycle was used for representation. The CV graphs were then traced in a way to use Cl_2_/Cl^–^ equilibrium potential as a reference: a tangent to the oxidation signal of Cl^–^ is traced in the part where the current density is superior to 100 mA/cm^2^. Then, the voltammogram is displaced in a way such that the tangent intersects the abscises in 0. This process allows for the comparison of CV despite possible instabilities of the Ag/AgCl reference. Moreover, it allows us to compare the CV curves from different studies that use other reference electrodes. The non-coincidence of Mg/Mg^2+^ peaks between different CV measurements (or cycle) can be a consequence of salt composition variations as the experiment progresses. 

The materials used in this study are two austenitic steels provided by ORNL with a 4 wt% of aluminium. Their chemical compositions communicated by the provider are given in Table 1. OC1 was preoxidised under dry air for 24 h at 700 °C, and OC4 was preoxidised under dry air for 24 h at 800 °C [26]. The alloys were preoxided on a silica rack placed in the oven. They were hanged by an alumina stem. They were inserted when the oven was at preoxidation temperature. The samples were cooled in the oven. They were immersed, as shown in Figure 1b.

The outlet gas was analysed during some experiments using a Pfeiffer Vaccum (Annecy, France) Omnistar GSD 301C mass spectrometer with a 950 V coil voltage.

Raman spectroscopy was performed on preoxidised alloys using a HORIBA (Palaiseau, France) LabRam HR800 with 532 nm laser.

Thermo-gravimetry analysis (TGA), using a Setaram (Caluire, France) TAG 24 under sweeping argon with a heating rate of 2 °C/min, coupled to mass spectrometry, was performed on the salt in order to identify the salt dehydration temperatures and the species formed during this process. 

The coupons were observed using a Zeiss (Rueil-Malmaison, France) Gemini 2 Crossbeam 550 FIB-SEM and a Zeiss Gemini Ultra 55 SEM coupled with a Brucker (Palaiseau, France) Nano EDX detector. After these preoxidations, OC1 and OC4 samples were immersed in molten NaCl-MgCl_2_ at 600 °C for 500 h. They were half-immersed in the salt and hanged with a silver wire. Each alloy was immersed in its own reactor to prevent any interaction between the different coupons. The potential of each coupon was measured during the corrosion test.

## 3. Results

### 3.1. NaCl-MgCl_2_ Purification Process

#### 3.1.1. Dehydration of the Salts

**Figure 2:**
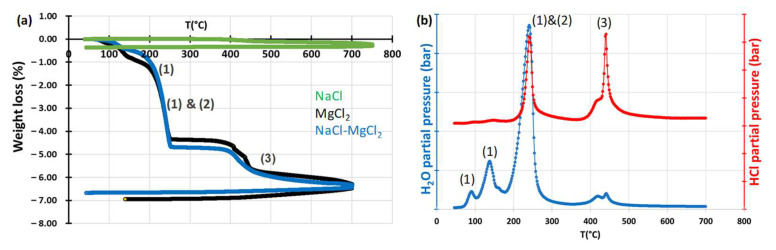
(**a**) Weight loss as a function of time of pure NaCl in green, pure MgCl_2_ in black and NaCl-MgCl_2_ in blue. (**b**) Partial pressure as a function of time of H_2_O in blue and HCl in red during NaCl-MgCl_2_ dehydration.

The melting process of NaCl, MgCl_2_, and NaCl-MgCl_2_ eutectic were monitored using TGA experiments coupled with a mass spectrometer to analyse the outlet gas. Figure 2 shows the mass loss and the mass spectrometer analysis of the salt sample. The heating rate was 2 °C/min. Results are presented in %wt loss to allow the comparison between different masses of salts. The productions of H_2_O and HCl are detected on the mass spectrometer. In Figure 2b, the blue curve corresponds to H_2_O partial pressure and the red curve to HCl partial pressure. According to the literature, MgCl_2_ is very hygroscopic and hydrolyses during the melting process, leading to the formation of MgOHCl (reaction (2) in Figure 2a)) as an intermediate product and MgO (reaction (3) in Figure 2a)) as a final product [7,27,28]. The competition between hydrolysis (reaction (2)) and dehydration (reaction (1)) can be observed at 250 °C, where the two reactions occur according to Figure 2b.

(1) $${\mathrm{MgCl}}_{2}\cdot H_{2}O\to {\mathrm{MgCl}}_{2}+H_{2}O\;$$

(2) $${\mathrm{MgCl}}_{2}\cdot H_{2}O\to \mathrm{MgOHCl}+\mathrm{HCl}\;$$

Only hydrolysis is detected at 450 °C with the second peak of HCl and corresponds to reaction (3).

(3) MgOHCl→MgO+HCl

These results are in accordance with the literature [7,27,28]. According to the mass loss due to reaction (3), 113.7 mg of NaCl-MgCl_2_ led to the formation of 1.44 mg of MgO. Assuming that the same proportions are present in a 182 g batch of salt for an immersion experiment, the expected MgO quantity is 2.30 g. As the solubility limit of MgO is approximately 2 × 10^−3^ mol/L at 600 °C, the salt is over-saturated in oxide ions after melting [28].

To limit the hydrolysis, the salt was dehydrated at 120 °C during 48 h and then melted at 600 °C.

**Figure 3:**
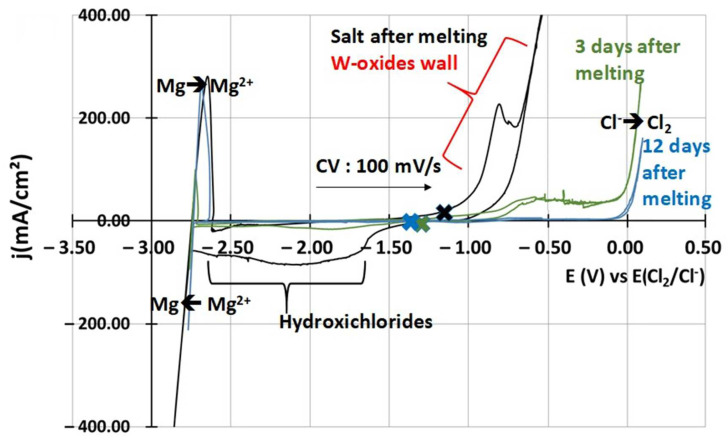
j-E curves obtained by cyclic voltammetry of NaCl-MgCl_2_ during the purification process. The black curve corresponds to a CV after the melting of the salt, the green curve to a CV made 3 days after melting and the blue curve to a CV made 12 days after melting. Blue, green and black crosses correspond to the open circuit potential of both salts after 1 h at rest.

Figure 3 shows the j-E curves obtained by cyclic voltammetry before and after purification. High peaks of oxides and hydroxychlorides are observed just after melting [29]. Tungsten oxides correspond to the high oxidation signal circled in red between −0.5 V vs. E(Cl_2_/Cl^−^) and −1 V vs. E(Cl_2_/Cl^−^) that hides the chlorides oxidation signal [19]. Hydroxychlorides correspond to the reduction wave circled in blue between −1.6 V vs. E(Cl_2_/Cl^–^) and −2.5 V vs. E(Cl_2_/Cl^−^), according to Skar et al. [29]. After a few days, the hydroxychloride peak disappears, as one can see on the green curve. It is assumed that hydroxychlorides changed into MgO, according to reaction (3) [7].

The oxides’ signal also decreases as oxides likely precipitate into MgO. After 12 days, depending on the salt purity, no signal is observed between the reduction peak of Mg^2+^ and the oxidation peak of Cl^−^. This state is shown by the blue curve in Figure 3. During the purification, the open circuit potential of the salt, symbolised by the blue, green and black crosses on the j-E curves, shifted downwards.

#### 3.1.2. Salt Purification

Two purification methods were tested: (i) one is to electrolyse the salt on a glassy carbon electrode, with the objective to remove oxide ions by CO_2_ production; (ii) the second one is to perform pure argon sweeping above the liquid salt and wait for oxide precipitation in the argon environment.

**Figure 4:**
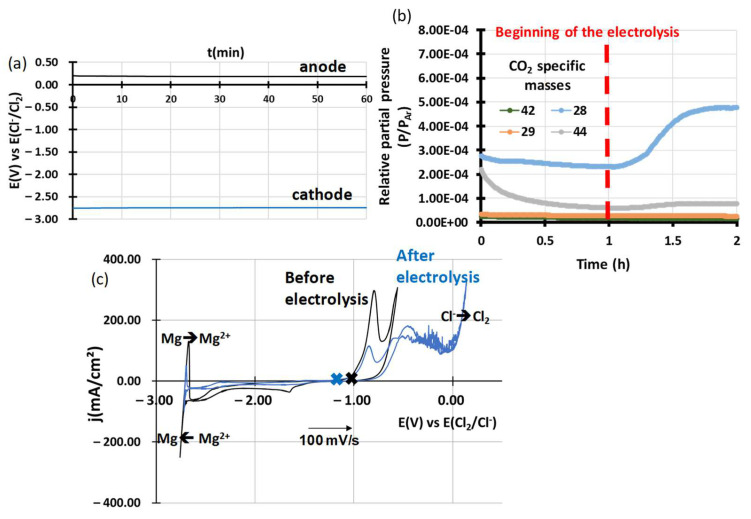
(**a**) Potential value of anode in black and cathode in blue during the 1 h electrolysis. (**b**) CO_2_ specific mass measurement obtained by mass spectrometer fixed at the gas outlet line. (**c**) j-E curves obtained by cyclic voltammetry of NaCl-MgCl_2_ before and after the first electrolysis. Blue and black crosses correspond to the open circuit potential of both salts after 1 h at rest.

Electrolysis on a 2 mm diameter glassy carbon anode provided by Goodfellow with a 1 mm diameter tungsten cathode was used to try to purify the salt faster. Electrolysis was conducted on a glassy carbon anode. The anode surface was 1.28 cm^2^, and the cathode surface was 0.66 cm^2^. The current density was 54 mA/cm^2^, and 256 C were delivered during the electrolysis. The value of the potential of the anode (WE) and cathode (CE) are reported in Figure 4a. They are constant; the anode value is E = 200 mV vs. E(Cl_2_/Cl^−^) and corresponds to a potential on the chloride oxidation signal, and the cathode potential is E = −2.75 V vs. E(Cl_2_/Cl^–^) and corresponds to the reduction of Mg^2+^ into Mg. During the electrolysis, a mass spectrometer was coupled to analyse the outlet gas. Figure 4b shows the analysis of the outlet gas during two hours: during the first hour, the salt was at rest, and during the second hour, the salt was electrolysed. During electrolysis, only CO_2_ was detected among O_2_, H_2_O, CO, CO_2_, HCl and Cl_2_. Cyclic voltammetry made before and after the electrolysis shows that the signal of the hydroxychloride (at E = −0.9 V vs. E(Cl_2_/Cl^−^)) almost disappears after the electrolysis [19,29]. The oxides’ first peak signal (at E = −1.6 V vs. E(Cl_2_/Cl^−^)) is also reduced [29]. Further electrolyses were made, but no gas production and no diminution of oxide peaks were observed, whereas a slight diminution of the hydoxychlorides was still observed. This might be the sign that the precipitation of MgO is simultaneous with the reaction of the hydroxychlorides during the first electrolysis. However, the electrolysis had no impact on MgO. During the purification, the open circuit potential of the salt, symbolised by the blue and black crosses on the j-E curves, shifted downwards.

**Figure 5:**
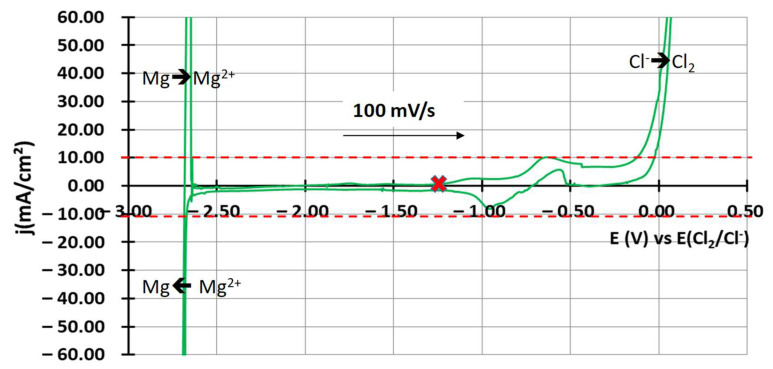
Cyclic voltammetry obtained after one week of pure argon sweeping at 600 °C. Red cross corresponds to the open circuit potential of the salt after 1 h at rest. Red dotted lines correspond to the 10 mA/cm² frame.

The second purification method is based on Ar gaseous sweeping above the molten salt. After one week with a flow rate superior to 20 L/h^−1^, the cyclic voltammogram in Figure 5 was obtained. MgO precipitation at the bottom of the crucible, identified by X-ray diffraction after salt solidification, occurred during Ar sweeping.

To obtain a CV identical to that shown in Figure 5, the electrolysis method requires one electrolysis, implying the formation of CO_2_ at the anode and Mg° at the cathode; then, wait 4 days instead of 12 days. In order to avoid the possible influence of electrolysis products (Mg° and dissolved CO_2_) on the salt, the method with Ar sweeping was preferred. To conclude, the reference state to start corrosion experiments is estimated by cyclic voltammetry and presented in Figure 5: the absolute value of the current density has to be lower than 10 mA/cm^2^. This current density ensures that the MgO concentration in the salt is inferior to 200 ppm, as shown by Skar et al. [29]. This was the best compromise between the purity of the salt and the time to reach this purity level. However, other criteria could be chosen. There are still oxidation and reduction signals between −1 V vs. E(Cl_2_/Cl^−^) and −0.5 V vs. E(Cl_2_/Cl^−^). They very likely correspond to various tungsten oxides, as shown by Chmakoff et al. [19].

### 3.2. OC1 and OC4 Corrosion

#### 3.2.1. Alloys, Microstructures and Preoxidations

**Figure 6:**
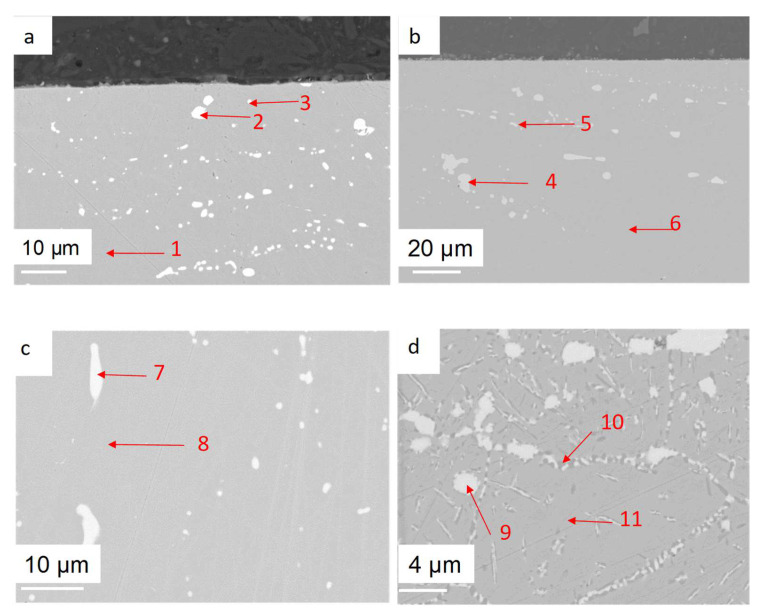
SEM-BSE cross-section pictures of OC1 and OC4. (**a**) OC1 before preoxidation, (**b**) OC1 after preoxidation under dry air for 24 h at 700 °C, (**c**) OC4 before preoxidation, (**d**) OC4 after preoxidation under dry air for 24 h at 700 °C. Numbers and arrows correspond to the EDS spot analysis.

Both alloys were ground with P1200 SiC paper. During the preoxidation, the microstructure of OC1 did not change, as shown in Figure 6a,b. It is composed of a matrix that contains Nb-rich precipitates (analyses spec. 2 and 3 in Table 2). There are large precipitates and small precipitates that do not have the same composition, as one can see comparing spec. 2/3 or spec. 4/5 in Table 2. The EDS analyses are semi-quantitative, and their accuracy is guaranteed by the %wt sum, which is close to 100%.

Their microstructures were studied using SEM-EDS (Zeiss & Bruker, Palaiseau, France). The microstructure of OC4 evolved as the Nb-rich precipitates (analysis spec. 7 in Table 2) dissolved to form a Nb-rich (2.7 mol% Ni, 57.1 mol% Nb, 1.1 mol% Fe) phase that appears white and a Ni–Fe–Al-rich (34.5 mol% Ni, 28.0 mol% Fe, 24.9 mol% Al) phase that appears in the darkest contrast on Figure 6d). These phases are inter and intragranular: within the grain, they form acicular precipitates. Otherwise, they follow the grain boundaries. The compositions of these phases are given in Table 2, spec. 9, 10, 11.

Figure 6d shows that the two kinds of precipitates are present in the grain boundaries.

The equilibrium microstructure of these two alloys was calculated using Thermocalc 2020a and TCFE8 database at 600 °C. The phases predicted by the software and presented in Table 3 and Table 4 do not correspond to the phases observed with the SEM-EDS. This might mean that the microstructure of both alloys is out of equilibrium before the corrosion test. Thermocalc predicted only two phases among all that were observed: the austenitic matrix, denoted FCC_A1#1 in Table 3 and Table 4, and niobium carbide that corresponds to spec. 9 in Table 2 or to FCC A1#2 in Table 4. Results are the same for simulations made at preoxidation temperatures (700 °C for OC1 and 800 °C for OC4).

**Figure 7:**
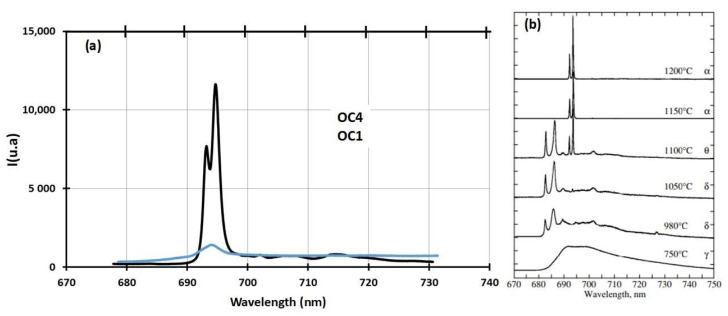
(**a**) Raman spectra of OC1 and OC4 after preoxidation. (**b**) Reference alumina Raman spectra.

The alumina scale was characterised using Raman spectra. The results are shown in Figure 7a and compared to reference spectra in Figure 7b [30]. A high-intensity signal at a wavelength equal to 695 nm was observed for preoxidised OC4, and a low-intensity signal was observed at the same wavelength for preoxidised OC1. These signals correspond to Cr^3+^ luminescence as those ions are present within the Al_2_O_3_ structure [30]. This means that the alumina scale is much thicker on OC4. This is likely due to the higher temperature of its preoxidation. According to the literature, α-alumina grew on OC4, and γ-alumina grew on OC1 during the preoxidation [30]. The slight shift between the experimental spectra and the reference ones is due to the zeroing procedure of the instrument. 

#### 3.2.2. Dissolved Species in NaCl-MgCl_2_ after Corrosion

**Figure 8:**
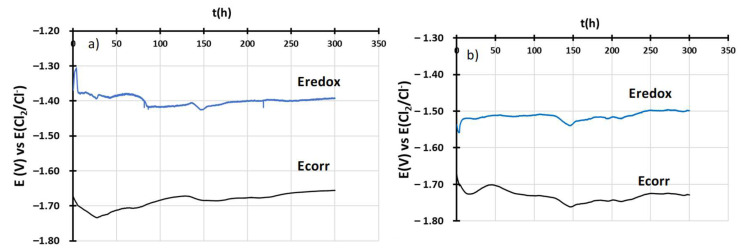
E-t curves of alloys during immersion (**a**) OC1 (**b**) OC4. Black curves correspond to Ecorr and blue curve to Eredox.

During the first 300 h of immersion, the E-t curves at i = 0 mA were recorded and are presented in Figure 8. The Ecorr (black curve) is stable for both alloys but the OC4 potential is a bit lower than OC1, which signifies that OC4 should corrode less. For both measurements, the Eredox measured on the W electrode is stable during the immersion.

**Figure 9:**
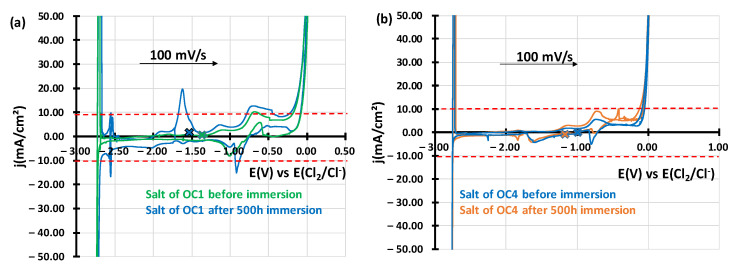
j-E curves obtained by cyclic voltammetry of (**a**) OC1 and (**b**) OC4 salts before and after corrosion at 600 °C. Blue, orange and black crosses represent the open circuit potential of the salts after one hour at rest. WE and CE are in tungsten.

At the end of the corrosion test, CVs were performed using the same electrodes and parameters as presented above. The results are presented in Figure 9. The signals of oxides between E = −1 V vs. E(Cl_2_/Cl^−^) and E = −0.2 V vs. E(Cl_2_/Cl^–^) have increased in OC1 salt, and an oxidation peak at E = −0.7 V vs. E(Cl_2_/Cl^–^) has increased in OC4 salt. These variations can be attributed to a slight increase in oxygen or water from the environment. No dissolved elements were observed in the OC4-salt by CV and by ICP-AES. An oxidation signal was observed in the OC1 salt between E = −1.7 V vs. E(Cl_2_/Cl^–^) and E = −1.5 V vs. E(Cl_2_/Cl^–^).

**Figure 10:**
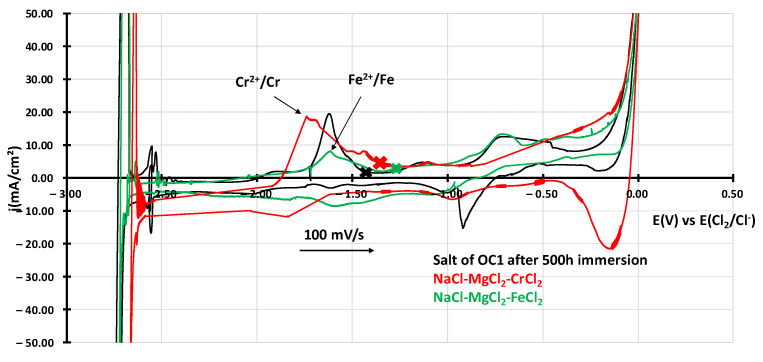
j-E curves obtained by cyclic voltammetry of OC1 salt in black compared with standard curves of NaCl-MgCl_2_-0.01 g FeCl_2_ in green and NaCl-MgCl_2_-0.11 g CrCl_2_ in red. Crosses correspond to OCP of the curve of its color.

According to standard curves presented in Figure 10 obtained by adding 0.01 g of FeCl_2_ or 0.11 g of CrCl_2_ in NaCl-MgCl_2_ at 600 °C, chromium and iron could have dissolved in the salt as the peak observed in OC1-salt is just between the peaks of Cr/Cr^2+^ and Fe/Fe^2+^ oxidation peaks. 

ICP-AES measurements in Table 5 show a similar concentration of iron and chromium in the salt, where OC1 was immersed, contrary to the salt of OC4, where no dissolved metals were identified. This confirms the trend observed on the voltammograms. However, the concentrations measured by ICP-AES were slightly lower than the ones measured by cyclic voltammetry (2.4 × 10^–3^ mol·L^–1^ for Cr and 1.2 × 10^–3^ mol·L^–1^ for Fe). 

#### 3.2.3. Corrosion of OC1 in Liquid Salt

**Figure 11:**
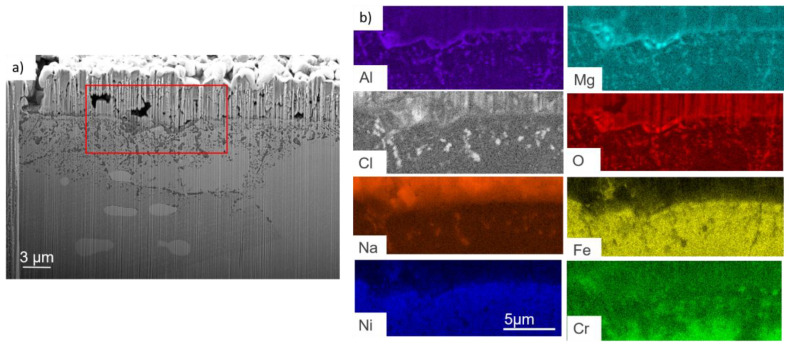
(**a**) FIB-SEM observation and (**b**) EDS-mapping of the zone presented by the red rectangle (**a**) of OC1 upper zone after corrosion.

The corroded sample was FIB-cutted and observed by SEM and analysed by EDS. The observation presented in Figure 11 shows a salt layer on top of the sample. At the interface of the alloy and salt, a Mg–Al–O oxide layer can be identified. Underneath, intragranular corrosion occurs. Mg–Al–O oxides, as well as some enrichment in chlorine, sodium and magnesium, are observed.

**Figure 12:**
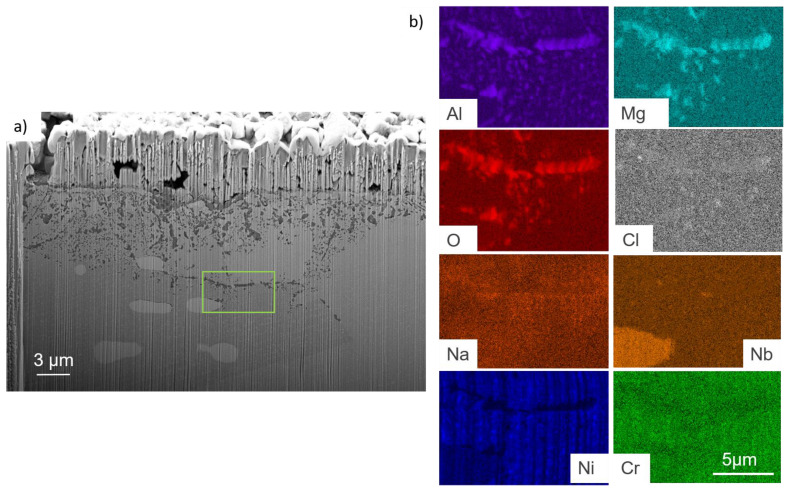
(**a**) FIB-SEM observation and (**b**) EDS-mapping of OC1 lower zone. Green box corresponds to the mapped area.

The lower zone of the sample was also analysed using EDS mapping at 15 keV, which is presented in Figure 12 and corresponds to the green rectangle. In the intergranular zones, Mg–Al–O oxides can also be detected, but the concentrations in chlorine and sodium are almost negligible. 

**Figure 13:**
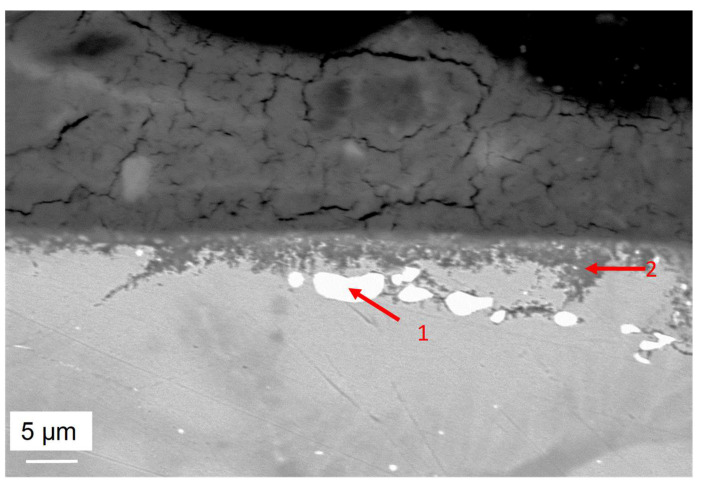
SEM-BSE observation of cross-section of OC1 sample oxidised 500 h at 600 °C in liquid salt. The compositions of EDX spec. #1 and #2 marked by the red arrows, are given in Table 6.

SEM observations show the same corrosion attacks in the inter and intragranular zones. The niobium precipitates are stable during the corrosion tests, as shown in Figure 13 in accordance with Table 2. The microstructure of OC1 did not evolve during the immersion test. It means that the kinetics for the microstructure change are too slow at 600 °C. EDS mapping of the corroded layer showed a limited dissolution of iron and chromium from the matrix, which is an encouraging result in comparison to what is usually observed in iron-based alloys. This reduced dissolution may be a consequence of the presence of an alumina scale.

**Figure 14:**
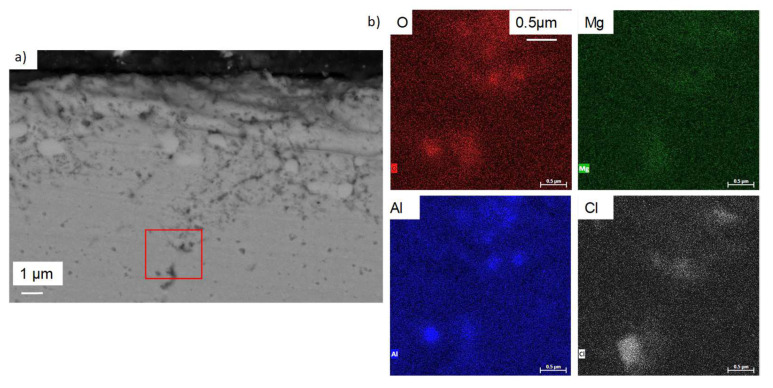
(**a**) SEM image of cross-section of OC1 sample corroded 500 h at 600 °C in liquid molten NaCl-MgCl_2_, (**b**) EDS mapping of intergranular corrosion corresponding to the red rectangle zone in (**a**).

EDS analyses of Table 6, spec. 2 show a Mg/Al ratio that corresponds approximately to MgAl_2_O_4_. Other metallic elements are detected. They are likely observed because they are present in the alloy around the oxide analysed in spec. 2. This could be a consequence of the oxide thinness, as the spec. is near the surface in a zone that corresponds to a grain boundary. The high percentage of oxygen does not correspond to MgAl_2_O_4_ but no other oxide exists. Therefore, it is very likely that MgAl_2_O_4_ forms at the upper parts of corrosion attacks. EDS mapping at 10 keV of intergranular corrosion of the red square zone in Figure 14b) shows that only Al–O–Cl are present at the tip of the internal oxidation zone. 

#### 3.2.4. Corrosion of OC1 under Gas Phase

**Figure 15:**
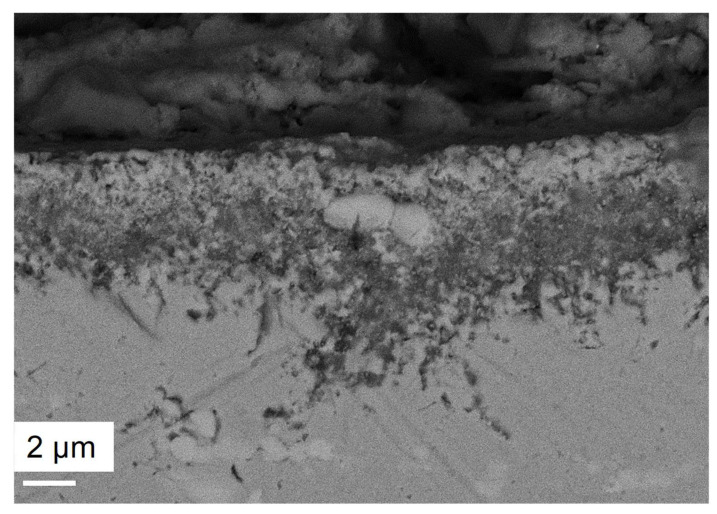
SEM-BSE image of cross-section of OC1 oxidised 500 h in the gas phase at 600 °C.

In the corrosion test under the gas phase, the corrosion facies are similar to the one observed under the liquid phase. An internal intragranular corrosion can be observed down to 2 µm below the alloy/gas interface in Figure 15. These internal corrosion products were analysed by EDX, leading to a molar composition of 8.5% Mg, 15% Al, 32% O, 26% Fe, 12% Ni and traces of other metals. It is, therefore, assumed that they are MgAl_2_O_4_ precipitates. Some grain boundaries are also corroded, but they are rarer than those in the OC1 part that are corroded in the liquid phase. 

**Figure 16:**
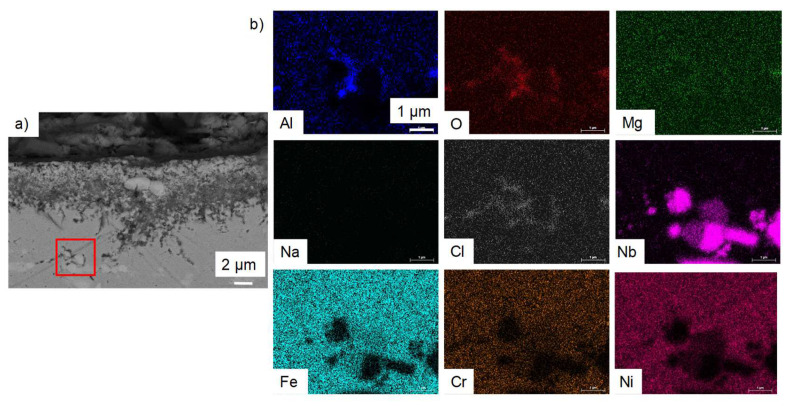
(**a**) SEM cross-section of OC1 gas corroded part and (**b**) red square corresponds to the EDX mapping of intergranular corrosion in OC1 gas-corroded part.

As in the liquid phase, only aluminium and oxygen are present at the bottom of the intergranular attacks, as shown by the EDS mapping performed at 10 keV (Figure 16b)) in the zone represented by the red square in Figure 16a. The intergranular attacks are less deep than in the liquid phase of the coupon. The corrosion products are similar to those in the liquid phase. Chlorine is present near the Al–O oxide around the niobium precipitates. There is no magnesium. Therefore, the oxide is likely Al_2_O_3_. Oxygen and chlorine seem to diffuse faster than magnesium.

#### 3.2.5. Corrosion of OC4 in Liquid Salt

**Figure 17:**
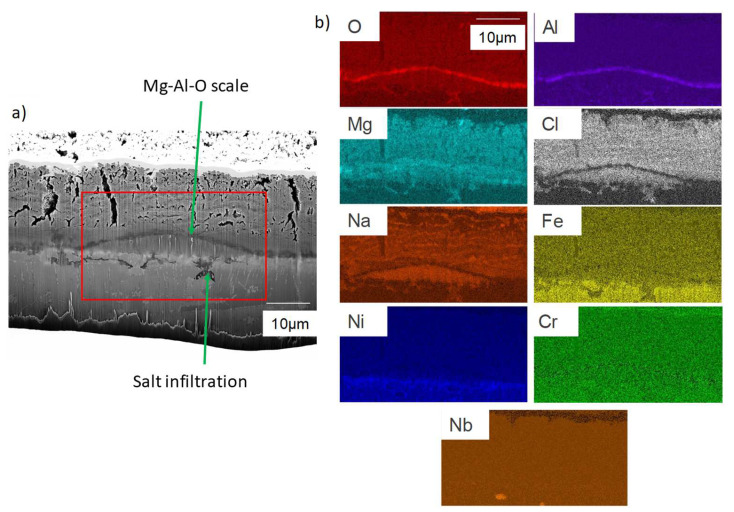
(**a**) FIB-SEM observation and (**b**) corresponding EDX-SEM mapping of OC4 immersed part. Red box corresponds to the mapped area.

OC4 sample was also FIB-cutted and observed at a 15 keV energy. Results are shown in Figure 17. FIB observations of corroded OC4 show a spalled Mg–A–O scale with salt beneath, as shown by the EDS mapping in Figure 17b. Infiltrations of Na–Mg–Cl occur in the matrix preferentially at grain boundaries.

**Figure 18:**
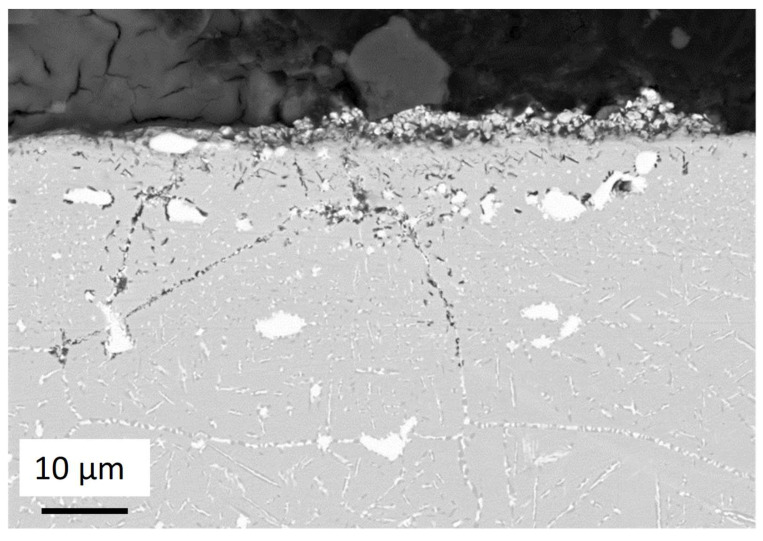
SEM observation of OC4 corroded in liquid phase.

SEM observation in Figure 18 shows that internal corrosion occurs at the grain boundaries. 

**Figure 19:**
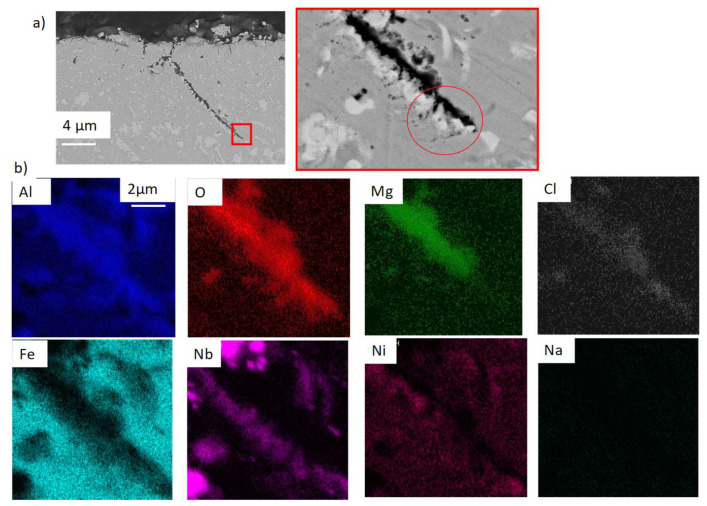
(**a**) SEM cross-section and (**b**) red square corresponds to the EDX mapping of intergranular corrosion in OC4 corroded in liquid phase. Red circle corresponds to the area where only Al and O are observed.

The corrosion is intergranular and 10 µm-deep on average. EDS analysis of Figure 19 shows that the internal precipitates are Mg–O–Al rich. According to the thermodynamic databases (HSC), the only existing mixed oxide is MgAl_2_O_4_. In the following, the presence of this oxide will be assumed. The Nb-rich carbides seem stable during the corrosion process. As in OC1, Al–O–Cl and no Mg are observed at the tip of intergranular precipitates, as shown by the red circle in Figure 19. The precipitation of Al_2_O_3_ could occur at the tip of the internal precipitation of MgAl_2_O_4_ where the (Fe, Nb, Ni) phase precipitates.

#### 3.2.6. Corrosion of OC4 under Gas Phase

**Figure 20:**
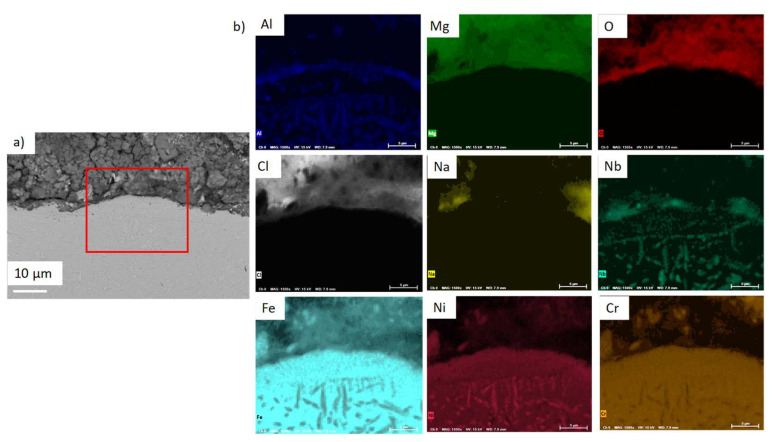
(**a**) SEM cross-section observation of OC4 gas corroded part. (**b**) Red square corresponds to the SEM-EDX mapped zone.

SEM image and SEM-EDX cartography of Figure 20 show that homogenous dissolution of the alloy occurs. At the top of the sample, a salt deposit is revealed in the darkish grey contrast. It corresponds to the condensed part of salt vapour. In this part of the sample, there is no preferential dissolution of any alloying element. A Mg–O–Al oxide seems to have formed at the top of the sample.

## 4. Discussion

During the preparation of the salt, it was observed in Figure 4 that only CO_2_ was produced. Therefore, the anodic reaction could be the oxidation of carbon reacting with oxide ions according to reaction (4).

(4) $$C+2O^{2-}={\mathrm{CO}}_{2}+4e^{-}$$

The cathodic reaction would be:

(5) $${\mathrm{Mg}}^{2+}+2e^{-}=\mathrm{Mg}$$

The global reaction during electrolysis would be (4) + 2(5):

(6) $$C+2O^{2-}+2{\mathrm{Mg}}^{2+}={\mathrm{CO}}_{2}+2\mathrm{Mg}\;$$

As observed on the cyclic voltammetry before and after the electrolysis in Figure 3, the reduction peak between E = −1.6 V vs. E(Cl_2_/Cl^–^) and E = −2.5 V vs. E(Cl_2_/Cl^–^) that Skar et al. associated with hydroxychloride reduction decreased after the electrolysis [29]. The electrolysis purified the salt faster by accelerating the disappearance of MgOHCl at the beginning of the process. At that time, all the MgOH^+^ is not yet transformed into MgO and can react during the electrolysis on the glassy carbon anode while magnesium is deposited at the tungsten cathode.

The anodic reaction proposed is then:

(7) $$2{\mathrm{MgOH}}^{+}+C={\mathrm{CO}}_{2}+2{\mathrm{Mg}}^{2+}+H_{2}+2e^{-}$$

The cathodic reaction is reaction (5). The global reaction is (7) + (5).

(8) $$2{\mathrm{MgOH}}^{+}+C={\mathrm{CO}}_{2}+{\mathrm{Mg}}^{2+}+\mathrm{Mg}+H_{2}$$

In this case, H_2_ production should be observed by the mass spectrometer. Until now, this mass has not been monitored during the electrolysis. Further work will attempt to observe H_2_ production to conclude the electrolysis reactions.

During the electrolysis, magnesium dissolution from the cathode was observed when the current was not high enough [31]. This can decrease the potential of the salt as Mg/Mg^2+^ acts as a buffer. This influences the corrosion behaviour of the materials in the molten salt. The electrolysis current density has to be chosen wisely. The current density used in this work was 54 mA/cm^2^ and was efficient. If it is too high, Cl_2_ or COCl_2_ can be produced, which is very dangerous.

The electrolysis reduces the amount of MgO present at the end of the purification as MgO is a reaction product from the hydrolysis of MgOHCl as shown in Figure 4 and reported by Kipouros et al. and Kirsch et al. [7,8]. The MgO already present does not react during the electrolysis. However, the remaining MgO present at the bottom of the crucible (identified by XRD analysis not shown here) could react with spots of the metal surface directly exposed to molten salts. To eliminate as much MgO as possible, the best solution is, after natural precipitation by argon sweeping (or electrolysis), to solidify the purified salt and cut out the bottom of the crucible where all the MgO is located.

According to Delpech et al. [32], the salt potential is controlled by Mg/MgCl_2_ and Cl_2_/Cl^–^ redox couples. In that case, the theoretical value for the salt potential is −1.33 V vs. E(Cl_2_/Cl^–^) at 600 °C, which corresponds to the measure made on the W electrode. As shown by Figure 8, the potential of OC4 and OC1 are inferior to that value. Therefore, corrosion is expected. Moreover, the results show that the salt potential can be controlled by influencing the activity of chloride ions or the activity of magnesium chloride. To mitigate corrosion in NaCl-MgCl_2_, the salt potential must be lowered by diminishing the activity of MgCl_2_ or by increasing the activity of Cl^–^. Diminishing the activity of MgCl_2_ would reduce the proportion of MgCl_2_ in the salt.

**Figure 21:**
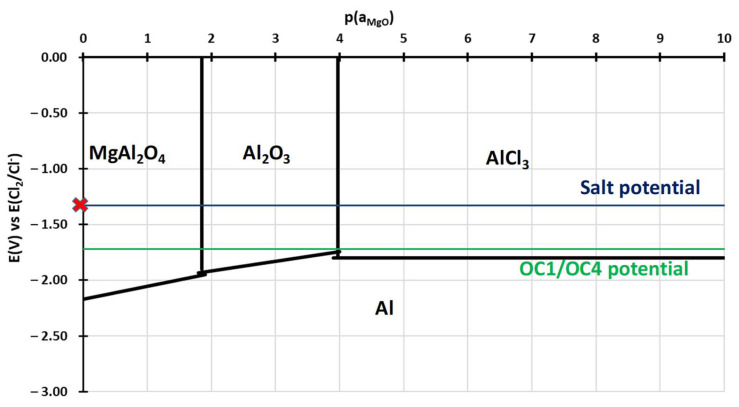
E-p(a_MgO_) diagram of aluminium in NaCl-MgCl_2._ Activity of Mg^2+^ equals 0.45 and Cl^–^ equal 0.5, according to Delpech et al. [32]. The Al activity equals 6.3 × 10^−7,^ and that of AlCl_3_ equals 1 × 10^−4^. All other activities equal 1. Red cross corresponds to the chemical conditions of the salt during immersion.

**Figure 22:**
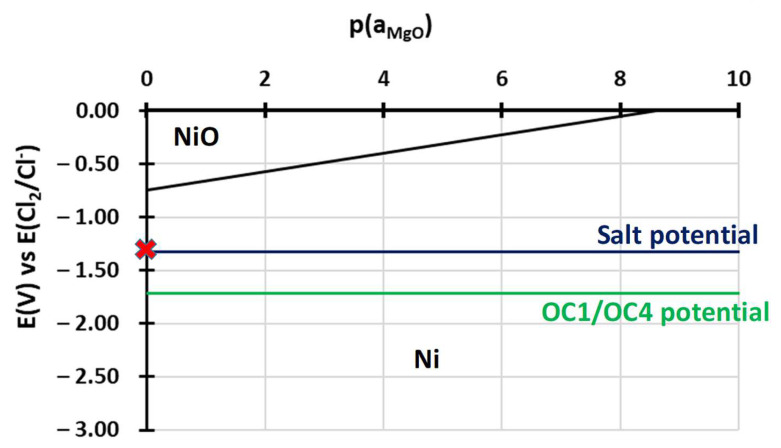
E-p(a_MgO_) diagram of nickel in NaCl-MgCl_2._ Activity of Mg^2+^ equals 0.45 and Cl^−^ equal 0.5, according to Delpech et al. [32]. The Ni activity equals 0.26, and that of NiCl_2_ equals 1 × 10^−4^. All other activities equal 1. Red cross corresponds to the chemical conditions of the salt during immersion.

**Figure 23:**
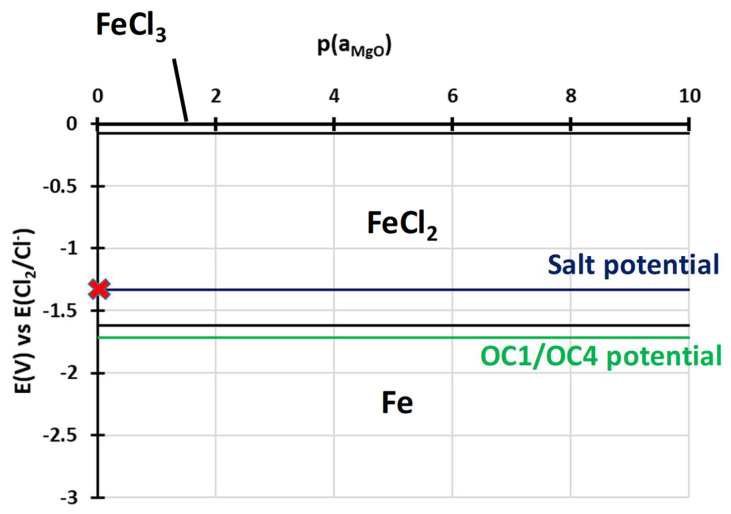
E-p(a_MgO_) diagram of iron in NaCl-MgCl_2._ Activity of Mg^2+^ equals 0.45, and Cl^−^ equals 0.5, according to Delpech et al. [32]. The Fe activity equals 0.69, and those of FeCl_2_ and FeCl_3_ equal 1 × 10^−4^. All other activities equal 1. Red cross corresponds to the chemical conditions of the salt during immersion.

**Figure 24:**
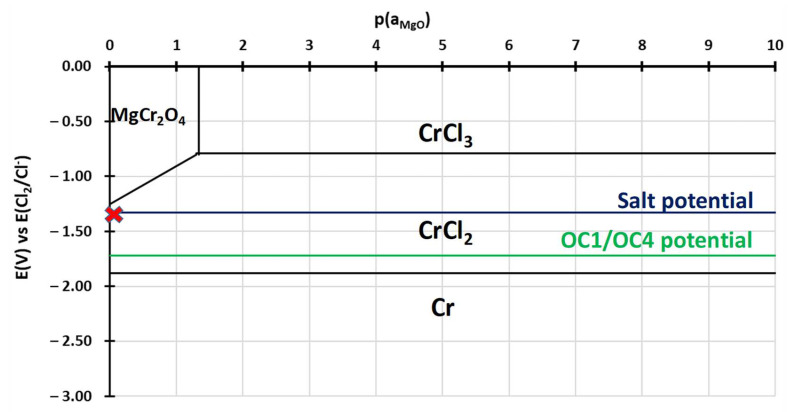
E-p(a_MgO_) diagram of chromium in NaCl-MgCl_2._ Activity of Mg^2+^ equals 0.45 and Cl^–^ equal 0.5, according to Delpech et al. [32]. The Cr activity equals 0.9, and those of CrCl_2_ and CrCl_3_ equal 1 × 10^–4^. All other activities equal 1. Red cross corresponds to the chemical conditions of the salt during immersion.

**Figure 25:**
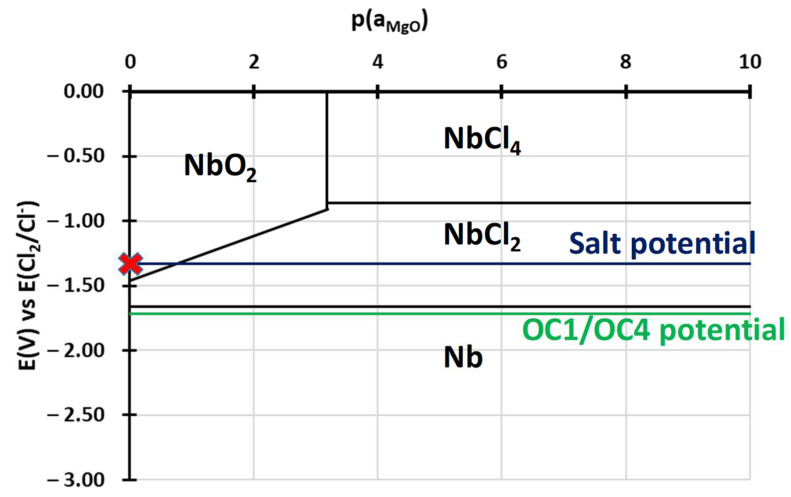
E-p(a_MgO_) diagram of niobium in NaCl-MgCl_2._ Activity of Mg^2+^ equals 0.45 and Cl^−^ equal 0.5, according to Delpech et al. [32] The Nb activity equals 2 × 10^−4^, and that of NbCl_2_ equals 1 × 10^−4^. All other activities equal 1. Red cross corresponds to the chemical conditions of the salt during immersion.

To understand the behaviour of the immersed alloys, E-p(a_MgO_) diagrams were traced. They are comparable to Pourbaix’s diagram: they describe the stability domain of metallic species as a function of the potential and the dissolved oxide ion activity. In this case, the dissolved oxide ion activity is represented by the MgO activity as oxide ions are not implemented in the thermodynamic databases (HSC, Factsage, Thermocalc). The diagrams are calculated with Cl^–^ activity equal to 0.5 and MgCl_2_ activity equal to 0.45, as calculated by Delpech et al. [32]. The activity of alloying elements is equal to the value calculated by Thermocalc in OC4 alloys at 600 °C: 0.26 for Ni, 0.69 for Fe, 0.9 for Cr, 6.3 × 10^−7^ for Al and 2 × 10^−4^ for Nb. The activities of dissolved species are considered equal to their concentration measured by ICP. As ICP values for the concentration of Cr, Fe, Ni and Al are between <8 × 10^−5^ and 2 × 10^−3^ mol/L, an activity equal to 10^−4^ is used for all dissolved Cr, Al, Ni, Fe chlorides as they are not observed in the OC4 salt. The value of 10^−4^ mol/L corresponds to the limit concentration given by the 10 mA/cm^2^ on the E-j curves that correspond to the background noise. All other activities are considered equal to 1 for the calculation of the predominance diagram. The Gibbs energies were calculated using the HSC database. It must be mentioned that thermodynamic data are inconsistent concerning the molten chloride environment, resulting in discontinuities in some diagrams. However, the E-p(a_MgO_) can provide a qualitative idea of the species that can form and is useful as a first approach when compared to the potential of the salt and of the alloys during the corrosion test. Due to their similar composition, OC1 and OC4 diagrams are very similar. That is why only OC4 diagrams are presented. The salt chemical conditions, in terms of E_salt_-pa_MgO_, are represented by the red cross in Figure 21, Figure 22, Figure 23, Figure 24 and Figure 25 that corresponds to E = E_salt_ and to a saturated salt in MgO (pa_MgO_ = 0), as solid MgO is observed at the bottom of the crucible. The aluminium diagram shows a large stability domain for corrosion products (Al_2_O_3_ and MgAl_2_O_4_) at the salt potential. The alumina scale should be stable and protective if the p(a_MgO_) conditions are convenient, i.e., for activity higher than 10^−5^. This shows the potential benefit of alumina-forming alloy in a molten salt environment. According to Figure 21, for a low oxide ion activity, the acid reaction occurs, dissolving Al_2_O_3_ into Al^3+^. In the present case, where MgO activity equals 1 and according to Figure 21, the basic reaction transforms Al_2_O_3_ into MgAl_2_O_4_. This is exactly what can be seen in Figure 14, Figure 16 and Figure 19, where alumina seems to be formed in the deepest part of the alloy before transforming into MgAl_2_O_4_. For instance, the circled zone in Figure 19 corresponds to a zone where alumina is present, while the zone just above it has already been transformed into spinel. Consequently, MgO activity is equal to 1 at the oxidised alloy/salt interface and decreases with depth in the alloy.

At the salt potential, Figure 22 shows that Ni is in its immunity domain. NiO can be formed for higher potential high-oxide activity conditions.

According to the iron stability diagram (Figure 21), iron should dissolve in Fe^2+^ form as the potential-oxoacidity of the salt is in the FeCl_2_ stability domain.

Chromium (Figure 24) and niobium (Figure 25) have similar behaviours as iron, but they can form oxides in higher potential/high MgO activity conditions.

According to their E-p(a_MgO_) diagrams, Nb is supposed to form NbO_2_ and Cr to form Cr^2+^.

These predictions are in accordance with what is observed in the case of OC1: chromium and iron dissolution are observed as expected (ICP-AES analysis of salt after OC1 corrosion test, Table 5). In the case of OC4, no iron nor chromium dissolution is observed. It is probably due to the thicker and then more protective alumina layer on the surface, as shown in Figure 7. In both cases, Nb does not oxidise. As it is present in the form of carbides, it would be necessary to study the thermodynamic stability of Nb carbides in the presence of salt.

Finally, the corrosion potentials of the OC1 and OC4 alloys can also be explained by the predominance diagrams, which show that they are close to the Cr/Cr^2+^ and Al/Al^3+^ thermodynamic equilibrium potentials (Figure 21 and Figure 24).

The two alloys have a complex microstructure containing several types of precipitates. The alloys are out of equilibrium, as suggested by the fact that many phases predicted by ThermoCalc were not observed. Predictions were made both at 600 °C and at the preoxidation temperature for both alloys (700 °C for OC1 and 800 °C for OC4), and the results did not correspond to the observations (see Table 3 and Table 4).

In general, OC1 and OC4 performed better than other iron-based alloys as the corrosion depth is less than 12 µm after 500 h of corrosion in liquid molten NaCl-MgCl_2_. Iron-based alloys and sometimes nickel-based alloys usually show corrosion that is several decades of micrometres deep [6,15,17]. The salt purification process also played a role in this result. Indeed, without any salt purification and if the immersion begins too soon, MgOHCl concentration cannot be neglected, leading to chlorhydric acid production by hydrolysis, as shown in Figure 4b. One could suppose it would lead to greater corrosion as H^+^ is an oxidising ion. The preoxidation and the presence of the alumina scale seem to improve the alloy’s resistance to corrosion because the alumina acts as a protective layer. It reacts with dissolved MgO, delaying the onset of the reaction between the salt and the alloy itself. Longer-term experiments would be needed to assess the protective role of the alumina scale.

In both alloys, two types of corrosion processes are observed during immersion. The first type is intragranular and is 2 µm deep on average. This intragranular corrosion is uniform for both alloys but is less pronounced for OC4. (See Figure 13 for OC1 and Figure 18 for OC4). As shown by the CV (Figure 9) and ICP analysis (Table 5) carried out after the immersion, the dissolution of iron and chromium are measured in the case of OC1 but not in the case of OC4.

No dissolved metals were measured on the CV (Figure 9) and ICP (Table 5) of the salt of OC4 after immersion. After pre-oxidation, the Raman signal (Figure 7) associated with α-alumina is much more intense in the case of OC4. The matrix of OC4 could not dissolve due to a thicker α-alumina scale, as observed on the Raman spectra. For both alloys, the alumina scale formed during the pre-oxidation (Figure 7) reacts with dissolved MgO to give a MgAl_2_O_4_ spinel (Figure 14 and Figure 19), as predicted by the E-pO^2–^ diagram of aluminium (Figure 21). The pre-oxidation enhanced the corrosion resistance of OC4 alloy, contrarily to the prediction of the chromium and iron stability diagram (Figure 23 and Figure 24); there is no depletion of these two elements in the corroded material, as Figure 17 shows. This can be attributed to the presence of the alumina scale that later transforms into MgAl_2_O_4_ and that acts as a protecting barrier, preventing the Fe and Cr from dissolution.

In both alloys, the deepest corrosion attacks are intergranular and are 10 µm deep on average. In the literature, intergranular corrosion is often observed because diffusivities of species are often higher in grain boundaries than in the alloy bulk. If the grain boundaries of the alloy contain precipitates (carbides, Ni_3_Nb, etc.), two processes may occur: the precipitate at the grain boundary corrodes if it is less noble than the matrix; otherwise, the matrix corrodes around the nobler precipitate. As shown in Figure 13 and Figure 18, for both alloys, in the grain boundaries, the matrix is oxidised around the Nb-rich precipitates. This is coherent with the fact that niobium has a high nobility in molten salts and should form NbO_2,_ according to Figure 25 [33]. This oxide could form around the precipitate, and the less noble matrix corrodes around it. The greyish precipitates in Figure 6d, whose composition is Ni–Fe–Al rich (Spec. 10 in Table 2), could be the one that corrodes in the grain boundaries. In that case, Al oxidises into Al_2_O_3_, as predicted by the stability diagram (Figure 21), and Fe and Ni diffuse in the matrix.

As shown on the EDX mapping in Figure 19, Al_2_O_3_ is observed at the tip of the MgAl_2_O_4_ precipitation. Mg is not detected at this tip, whereas Cl is detected. It means that Al_2_O_3_ forms first by faster diffusion of O and Cl and then transforms into MgAl_2_O_4_ with Mg diffusion. These observations lead to the hypothesis that the corrosion rate in those two alloys is controlled by oxygen and Cl diffusion. In the case of O diffusion, the corrosion kinetics would be parabolic, following Wagner’s law. The depth of corrosion can be expressed as:

$$X=\sqrt{2\frac{NoDo}{\nu N_{Al}}}t$$

where

*X* is the corrosion depth in cm, t is the time of immersion in seconds;

ν is the oxide molar ratio, which equals 1.5 in the case of Al_2_O_3_;

*N_Al_* is the molar fraction of aluminium in the alloy;

*NoDo* is the oxygen permeability in cm^2^/s.

According to Prilleux [34], work on oxygen permeability in iron-based alloys, the oxygen permeability at 600 °C in a Fe–20 Ni (%wt) alloy is 1.67 × 10^–15^ cm^2^/s for the intragranular zones. Under the hypothesis that the value is the same for OC1 and OC4 alloys that contain, respectively, 20%wt and 25%wt of nickel, the corrosion depth should be 2.6 µm, which is in good agreement with the observations of internal intragranular oxidation. For the intergranular zones, Sanviemvongsak [35] showed that oxygen permeability is 10 times higher in grain boundaries than in the matrix for the IN718 alloy. Assuming that the same ratio exists in OC1 and OC4 alloys, the intergranular corrosion should be 8 µm deep, which is close to what is observed. From these observations and calculations, it can be assumed that the internal oxidation could be controlled by oxygen diffusion.

The corrosion mechanism can be described as follows.

MgO, dissolved in the salt, reacts with the outer alumina scale to form MgAl_2_O_4_. Meanwhile, the iron and the chromium contained in the matrix dissolve into the salt (mainly for OC1), and the intragranular internal oxidation is controlled by O diffusion in the alloy. In parallel, O, Cl and Mg diffuse in the grain boundaries. O and Cl diffuse faster than Mg, and Al_2_O_3_ forms first at the tip of the intergranular oxidation. Then, Al_2_O_3_ transforms into MgAl_2_O_4_ when Mg reaches those regions. The Nb-rich precipitates do not react with the salt as they are protected by the nobility of Nb. TEM analysis at the matrix Nb precipitate interface would determine if the precipitates are protected by Nb oxide.

In the gas phase, a small layer of salt deposits on the coupon. The salt deposited must be liquid or nearly liquid as the temperature above the molten salt is likely superior to 500 °C. The deposited salt absorbs oxygen and moisture from the sweeping argon, and then MgO deposits on the coupon. The corrosion mechanism is similar to the liquid phase in the case of OC1, with a faster diffusion of oxygen that controls the corrosion mechanism. Inter and intragranular corrosion occurs with the formation of Al_2_O_3_ that later transforms into MgAl_2_O_4_.

Under the gas phase, OC4 seems very resistant, with no intergranular corrosion and no metal depletion. This performance seems to be a consequence of its better preoxidation, as a Mg–Al–O oxide that could be protective is observed.

## 5. Conclusions

This study showed that alumina-forming alloys are a promising alternative as a structural material in the design of an MSR. OC1 and OC4 should be improved to prevent their intergranular corrosion.

1. The purification protocol allowed us to control the purity of the salt and to obtain reproducible corrosion results. Pre-oxidation of samples in dry air led to alumina formation at the sample surface. For both alloys, Al_2_O_3_ formed during the pre-oxidation.
2. Alumina seems to be protective in the first steps of oxidation but becomes less efficient as it transforms into MgAl_2_O_4_. This behaviour can be explained by the stability diagram calculated considering the activities of elements within the steels. The predictions of the diagrams are in accordance with experimental observations. They could be used to design alloys a priori in order to make them resistant to molten salt corrosion.
3. For both alloys, Al_2_O_3_ formed during the pre-oxidation transformed to MgAl_2_O_4_, and internal intergranular corrosion occurred leading also to the formation of MgAl_2_O_4_ as observed on SEM cross-sections. At the internal corrosion front, Al_2_O_3_ precipitates were observed below the MgAl_2_O_4_ spinel precipitates, implying that Al_2_O_3_ is formed first and then transforms into the spinel. This mechanism occurs for both inter and intragranular corrosion. The internal oxidation depth is consistent with the diffusion of oxygen in the material, both in the matrix and in the grain boundaries, according to Wagner’s model. The diffusion of oxygen could, therefore, be the kinetically limiting factor. To sum it up, oxygen diffuses faster than Mg in the grain boundaries, leading to the formation of Al_2_O_3_, which later transforms into MgAl_2_O_4_. Due to the formation of a thicker alumina scale during the pre-oxidation, OC4 alloy performed better than OC1 with less intragranular corrosion.
4. Electrochemical techniques enable better control of the molten salt environment so that comparable results can be obtained in different batches of salt. They also enable a better understanding of the corrosion mechanism by providing information on the elements dissolved in the salts. Cyclic voltammetry allowed us to observe dissolved chromium.

To better understand the corrosion mechanism and observe the effectiveness of alumina, longer immersions are necessary. Corrosion kinetics will be necessary using immersions up to 5000 h in order to: (i) Assess the possible existence of a transitory step in the corrosion process. (ii) Determine the limiting step in the corrosion process. Moreover, studying OC1 and OC4 corrosion processes under various oxoacidity conditions by purifying the salt under the MgO solubility limit will be necessary. OC1 and OC4 should be improved to prevent their intergranular corrosion. This could be achieved by changing their composition or by optimising their heat treatment to favour the Nb-rich phase that does not corrode, as well as growing a thicker alumina scale on the two alloys. At the end of their service life, they would be considered nuclear waste due to their contact with NaCl-MgCl_2_-PuCl_3_. Considering the actual legislation on such waste, they could not be recycled. That being said, iron has a higher recyclability rate than nickel, advocating for the use of stainless steel rather than nickel-based alloy. If those alloys were used in Concentrated Solar Power plants, they could be re-casted after being rinsed from molten chloride residues, as molten chloride salts are soluble in water.

## Figures and Tables

**Table 1:** Table of chemical compositions of OC4 and OC1 alloys.

wt%	C	Ni	Cr	Al	Nb	Mo	Ti	W	Fe
OC4	0.1	25	14	3.5	2.5	2	0.05	1	Bal.
OC1	0.1	20	14	3	2.5	2	0.05	1	Bal.

**Table 2:** SEM-EDX composition of the numbered spec. in Figure 6.

mol%	Fe	Ni	Cr	Nb	Al	Mo	W	C	Sum(%wt)
Spec. 1	57.8	20.2	14.0	18.2	4.4	0.5	0.4	0	103.0
Spec. 2	45.7	10.1	14.0	18.2	2.2	6.0	4.0	0	107.9
Spec. 3	12.3	3.8	7.5	72.3	0.0	3.8	0.09	0	95.8
Spec. 4	43.2	11.5	11.6	21.9	0.0	5.6	5.03	0	100.8
Spec. 5	8.5	3.0	3.6	82.8	0.0	2.0	0.2	0	97.3
Spec. 6	57.6	18.7	11.6	0.5	5.8	0.9	1.5	0	96.9
Spec. 7	1.1	2.0	2.13	88.8	0.37	3.9	1.74	0	99.2
Spec. 8	47.2	23.2	14.0	1.4	3.0	1.7	1.6	0	92.0
Spec. 9	1.1	2.7	2.9	57.1	0.2	2.1	0	0	98.1
Spec. 10	28.0	34.5	8.0	1.0	24.9	0	0.7	0	92.6
Spec. 11	55.3	22.5	17.1	0.1	3.8	1.3	0	0	88.8

**Table 3:** Name and compositions of the phases in OC1 as predicted by Thermocalc at 600 °C.

OC1 Phases (mol%)	Fe	Ni	Cr	Al	Nb	W	Mo	C
FCC_A1#1	53	23	14	7	0.08	0.5	0.6	5 × 10^–5^
BCC_A2	66	5	22	5	0.02	0.02	0.04	7 × 10^–6^
Laves phases C14	58	0.4	6	2 × 10^–5^	8	9	16	0
NbNi_3_	0	75	0	0	25	0	0	0
Sigma	45	3	44	6 × 10^–5^	3	0.9	5	0
FCC A1#2	0.002	0	0.04	0	53	3 × 10^–4^	0	46

**Table 4:** Name and compositions of the phases in OC4 as predicted by Thermocalc at 600 °C.

OC4 Phases (mol%)	Fe	Ni	Cr	Al	Nb	W	Mo	C
FCC_A1#1	51	24	14	7	0.08	0.03	0.6	7 × 10^–5^
Laves phases C14	55	0.4	8	2 × 10^–5^	5	12	15	0
NbNi_3_	0	75	0	0	25	0	0	0
Sigma	45	3	45	5 × 10^–5^	0.01	1	5	0
FCC A1#2	0.002	0	0.04	0	53	3 × 10^–4^	0	46

**Table 5:** Results of ICP analysis of OC1 and OC4 salts after immersion.

Sample	Cr (mol·L^–1^)	Fe (mol·L^–1^)	Ni (mol·L^–1^)	Al (mol·L^–1^)	Nb (mol·L^–1^)	Mo (mol·L^–1^)
OC1	4.0 × 10^–3^	2.5 × 10^–3^	<7.7 × 10^–5^	<1.7 × 10^–4^	<4.9 × 10^–5^	<4.7 × 10^–5^
OC4	<2.3 ×10^–4^	<2.1 × 10^–4^	<2.0 × 10^–4^	<4.4 × 10^–4^	<1.3 × 10^–4^	<1.2 × 10^–4^

**Table 6:** SEM-EDX analyses of annotated spec. 1 and 2 in Figure 13.

mol%	O	Al	Mg	Cl	Na	Nb	Ni	Cr	Fe	Mo	W	C
Spec. 1	5.5	0.9	0.0	0.3	1.0	16.6	9.0	10.7	39.2	4.8	2.5	9.7
Spec. 2	44.5	12.6	7.2	7.3	0.3	1.1	5.5	2.5	9.7	1.8	0.0	7.5

## Data Availability

The data presented in this study are available on request from the corresponding author due to industrial concerns.
